# The application of single-molecule optical tweezers to study disease-related structural dynamics in RNA

**DOI:** 10.1042/BST20231232

**Published:** 2024-03-27

**Authors:** Tycho Marinus, Toshana L. Foster, Katarzyna M. Tych

**Affiliations:** 1 Chemical Biology 1, University of Groningen, Nijenborgh 7, 9747 AG Groningen, The Netherlands; 2 Faculty of Medicine and Health Sciences, School of Veterinary Medicine and Science, University of Nottingham, LE12 5RD Loughborough, U.K.

**Keywords:** optical tweezers, RNA, single-molecule

## Abstract

RNA, a dynamic and flexible molecule with intricate three-dimensional structures, has myriad functions in disease development. Traditional methods, such as X-ray crystallography and nuclear magnetic resonance, face limitations in capturing real-time, single-molecule dynamics crucial for understanding RNA function. This review explores the transformative potential of single-molecule force spectroscopy using optical tweezers, showcasing its capability to directly probe time-dependent structural rearrangements of individual RNA molecules. Optical tweezers offer versatility in exploring diverse conditions, with the potential to provide insights into how environmental changes, ligands and RNA-binding proteins impact RNA behaviour. By enabling real-time observations of large-scale structural dynamics, optical tweezers emerge as an invaluable tool for advancing our comprehension of RNA structure and function. Here, we showcase their application in elucidating the dynamics of RNA elements in virology, such as the pseudoknot governing ribosomal frameshifting in SARS-CoV-2.

## Introduction

RNA is a dynamic and flexible molecule, whose single-stranded nature allows it to have both intramolecular base pairing and multifaceted intermolecular interactions, resulting in the formation of complex three-dimensional structures. The classical central dogma of molecular biology traditionally focuses on RNA as an information messenger for the synthesis of proteins, however, in the last few decades a myriad of additional functions have been discovered for RNA molecules [1]. From the total RNA mass, only ∼3–7% is used for protein synthesis. RNA which is not translated into proteins is generally called non-coding RNA (ncRNA). These ncRNAs can be categorised into three main groups, namely, ribosomal RNA (rRNA, 80–90%), transfer RNA (tRNA, 10–15%) and regulatory ncRNA (<1%) [2]. In this review, we will focus on regulatory ncRNA. Since the discovery of ncRNA, it has become apparent that a significant portion of ncRNA molecules play important roles in the development of diseases [3]. For many ncRNAs, the secondary and tertiary structure significantly dictates their function, therefore, modulating their function with small molecule therapeutics will provide opportunities to regulate numerous cellular processes [4]. However, RNA structures are not static, they can undergo conformational changes that influence their active function. The dynamics of these structural changes are regulated, for example, by RNA-binding proteins and post-transcriptional modifications, in order to control their functional modalities [5].

A plethora of experimental techniques have already been innovatively developed and applied to study the diversity of RNA functions and how they are dictated by both the structure and the dynamics of the RNA molecules [6]. Most existing experimental methods to study RNA structure are the conventional approaches akin to those used to study protein structure, such as X-ray crystallography, small-angle X-ray scattering (SAXS), nuclear magnetic resonance (NMR) and cryo-electron microscopy (cryo-EM) [7]. It should be acknowledged that there is a disparity between the number of RNA-only structures available in online repositories such as the protein data bank (PDB) (1802 entries [8]) and those of proteins (187 344 entries at the time of writing, roughly 100-fold more [9]). This discrepancy is, in part, caused by the added complexity of studying RNA structure [7]. However, there are also several specialised methods that are unique to RNA [10], for example, chemical probing methods such as selective 2′-hydroxyl acylation analysed by primer extension (SHAPE) [11,12]. SHAPE is a common method to study RNA structure and can be used *in vivo* and *in vitro* [13]*.* By using chemical probing agents that only bind to flexible or unpaired bases of RNA molecules, SHAPE induces mutations in the resulting complementary DNA during reverse transcription of the target molecules, yielding mutational profiles, from which RNA structure can be deduced. However, neither chemical probing methods such as SHAPE, nor the aforementioned structural methods allow the direct real-time observation of large-scale structural dynamics which dictate RNA function. Furthermore, the mentioned techniques for probing RNA structure require the collection of data from large ensembles of molecules. This greatly limits the ability to observe infrequently occurring structures or transient intermediates, both important determining factors in understanding RNA function.

In contrast, single-molecule force spectroscopy using optical tweezers is a transformative methodology as it allows for the direct probing of time-dependent structural rearrangements of single RNA molecules [14]. Depending on the experimental setup, this technique presents unique versatility allowing for the exploration of diverse structural questions. An early example of optical tweezers to probe RNA structure was the study of the *add* adenine riboswitch, where optical tweezers were used to gain an in-depth understanding of structural changes in this riboswitch upon adenine binding [15]. More recently multiple research groups used optical tweezers to study the dynamics of an RNA structure, called a pseudoknot, which governs ribosomal frameshifting in SARS-CoV-2, a mechanism often exploited by viruses; for the translation of multiple unique proteins from of a single mRNA molecule [16–18].

As optical tweezers experiments can be performed under different conditions (e.g. using microfluidics) for the same molecule, structural changes can be correlated with diverse measurement conditions, e.g. different physiological conditions found in cells, by simulating cellular environments *in vitro* to better understand the impact of these structural changes. This renders optical tweezers an invaluable tool for the study of the intriguing world of RNA where environmental changes and the presence and absence of ligands, post-transcriptional modifications and RNA-binding proteins have large impacts on RNA behaviour [19]. In this review, we will briefly explain the foundational principles of optical tweezers experiments, and showcase instances where this technique has been used to progress the comprehension of RNA structure and function, particularly in the context of virus biology

## Optical tweezers for studying RNA structure

### Basics of optical tweezers experiments

Optical tweezers are rapidly evolving as a tool for conducting RNA structural studies. They typically provide nanometre length-scale resolution, pico-Newton (pN) force resolution and a time resolution of under 10 µs [20], allowing the step-by-step dissection of RNA structure and folding pathways. Notably, considering the characteristic contour length of a single nucleotide is ∼0.34 nm, where contour length is defined as the length at the maximum physically possible extension, and acknowledging that RNA folding kinetics are length-dependent, this technique can capture nuanced dynamics over very fast timescales, generally estimated within the range of 10–100 µs, particularly in the context of small hairpin loops [21]). An exemplary demonstration of these capabilities lies in the observation of single nucleotide elongation during RNA transcription [22].

**Figure 1.**
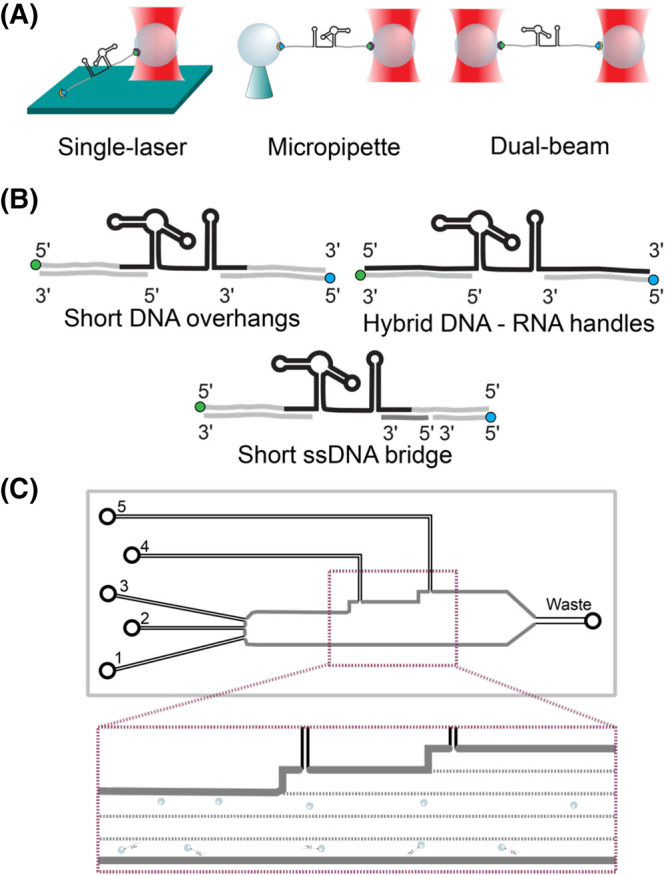
Overview of optical tweezers measurement setups. (**A**) Optical tweezer setup geometries. (**B**) RNA–DNA construct designs for measurement of RNA in the optical tweezers (RNA in black, DNA in grey). (**C**) A schematic of a microfluidic chip-based measurement setup, controlled laminar flow creates individual channels through the open chip design (black dotted lines indicate channel separation). Functionalized and non-functionalized beads (channel 1 and 3, respectively) are loaded in separate channels to be caught using the trapping laser.

There are many different optical tweezers experimental configurations for the characterisation of biological macromolecules on the single-molecule level; however, the general principles are analogous [20,23]. A common setup is one where a molecule of interest is tethered between 2 μm-sized beads. These beads are then caught in two laser beams, with alternative techniques using surface attachment or micro-pipettes to stabilise one of the beads (Figure 1A). Once the two beads with a molecule in-between them are entrapped between the two lasers, a force can be applied to the molecule by moving one laser away from the other. The resulting force response and length changes resulting from the stretching and unfolding of the molecule afford observation of these events at pN scale forces and nanometer-scale length changes.

Conducting optical tweezers measurements on any given RNA molecule, requires the tethering of the RNA between a stationary and a moving bead. To achieve this, DNA handles are linked to both 5′ and 3′ ends of the RNA molecule, forming an RNA–DNA hybrid duplex. Figure 1B shows a few example geometries depicting how RNA can be attached to DNA handles. These DNA handles are functionalised with biotin or digoxigenin moieties, which allows them to selectively bind to micron-sized biotin- or digoxigenin-coated beads, respectively [24]. While it is feasible to directly attach biotin or digoxigenin to RNA, most experiments use DNA handles since chemical modifications to DNA are easier. A consequential benefit of this approach is that there is additional separation created between the lasers and the RNA molecule of interest, thus mitigating damage to the RNA by heating or free radicals [25].

Following assembly of the RNA–DNA constructs, these constructs are incubated with either streptavidin or anti-digoxigenin beads. The loading procedure of the constructs into the optical tweezers varies for each setup. Commonly, the streptavidin and anti-digoxigenin beads are loaded on a microscopy slide. Within the optical tweezers, the lasers are then used to catch each type of bead. The beads are repeatedly brought into proximity until a signal with a specific force–distance response, indicative of the successful tethering of a sample between the two beads, is detected. Once a tether is formed, i.e. a single RNA–DNA construct is tethered between two beads, the measurement can be performed. Some optical tweezers have been equipped with microfluidics systems as illustrated in Figure 1C. In such cases, the channels can be loaded with different buffers and other samples, allowing for changes in measurement conditions in a single measurement chamber.

**Figure 2.**
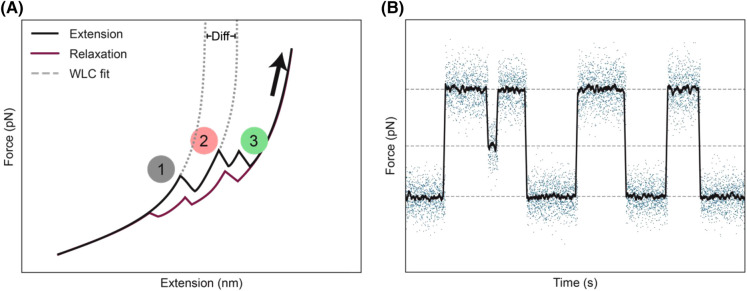
Examples of data that can be obtained from optical tweezers measurements. Schematic graph of: (**A**) Extension relaxation trace. During the extension phase (black line), three unfolding events are identified (numbered). The relaxation phase (purple line) shows the same three events, however, here the molecule refolds. Two worm-like chain (WLC) model fits are used to model the true length of the extension. This is done by calculating the difference of two WLC fits (shown as ‘Diff’). (**B**) Constant force trace, in blue the observed forces, black shows the sliding average. In this case there are three observable states indicated by the grey dotted line.

Three predominant measurement modes are routinely used in optical tweezers investigations: constant velocity, constant distance and constant force. The constant velocity measurement mode, where the RNA molecule is stretched and allowed to relax in repeated cycles, can be used to observe the full unfolding and refolding of an RNA molecule. In this mode, one bead is kept in place while the laser moves the other bead slowly away. This induces a force on the sample resulting in the stretching of the RNA–DNA construct followed by the unfolding of the secondary structural elements of the RNA molecule. These experiments will result in force–extension curves as depicted in Figure 2A which can be fitted with a worm-like chain model to obtain accurate length changes [26,27]. Unfolding events are identified as locations where the force drops coincide with extension increases, where each unfolding event results from the breaking apart of hydrogen bonds forming RNA secondary structure elements. Once fully unfolded, the beads are brought closer to each other again. During this relaxation phase, the secondary structure elements of the RNA molecule can re-form (as evidenced by the relaxation trace in Figure 2A). Since these are single-molecule experiments, by analysing the unfolding and refolding events of multiple individual RNA molecules, the resulting data provide insights into the probabilities of the existence of specific (un)folding patterns, while rarely occurring (mis)folded states are still detectable.

Conversely, the second and third measurement modes, namely contact distance and constant force, involve maintaining the beads either at a constant separation or with a sustained force between them. In these experiments, molecules are pulled to a specific distance, or force, often close to the equilibrium point between the folding and unfolding of a region of interest. Keeping the distance or force steadily at such a position allows the molecule to repeatedly switch from the folded to the unfolded state (Figure 2B). By combining the length, force and kinetics information, different (un)folding states can be identified.

The number of repeats for optical tweezers experiments is dependent on the dynamics of the targeted structure. In the case of a stable structure where only a single form is observed, as few as four molecules each unfolded and refolded 30 times would provide sufficient statistics. On the other hand, if multiple conformations of the structure are observed, the number of molecules measured and the number of repeats per molecule should be increased accordingly.

**Figure 3.**
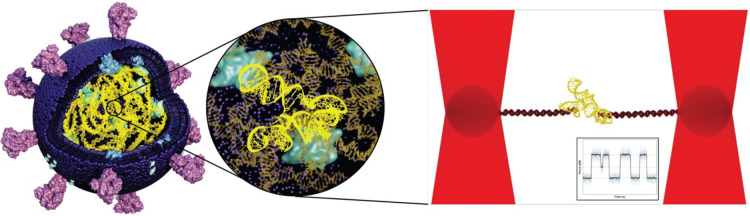
The use of optical tweezers to study viral RNA. Schematic of SARS-CoV-2 showing the spike proteins in light violet, the lipids in purple, RNA in yellow and other proteins in light blue and cyan; (zoomed image) highlight of the RNA pseudoknot structure; (right-hand panel) the same RNA pseudoknot in an optical tweezers setup, showing (inset) schematic data of its folding and unfolding dynamics. Image credit: Ana Radu, University of Groningen.

Together, optical tweezers-based methods enable the identification of distinct structural states of RNA molecules, and the direct real-time observation of transitions between these states. Here, we will give select examples that underscore the great potential of optical tweezers experiments in unravelling otherwise inaccessible details of RNA structural dynamics (Figure 3).

### Experimental examples

Viruses have highly diverse RNA structures; this diversity partially arises from the evolutionary pressure to constrain genome length [28]. This limitation has promoted the adaptation of RNA structure for a diverse set of alternative functions, spanning from orchestrating virion assembly to modulating viral protein levels [29,30]. This makes an in-depth understanding of RNA structural dynamics important for the development of antiviral compounds, given the pivotal role played by RNA structures in the interplay of viral life cycles and their multifunctional roles.

### The SARS-CoV-2 pseudoknot

The 2019 COVID-19 pandemic was caused by the severe acute respiratory syndrome-related coronavirus, a member of the coronavirus family characterised by a unique pseudoknot that induces a ribosomal frameshift [31]. Whilst pseudoknots are commonly found in RNA structures, this specific pseudoknot is unique to the coronavirus family and consists of three stem–loops instead of the conventional two. Mutational destabilisation of the pseudoknot has previously been shown to result in significant attenuation of the virus [32]. To investigate the structural and kinetic details of this pseudoknot, Neupane et al. [18] analysed its folding dynamics using optical tweezers.

Prior simulations and cryo-EM structural analyses of the SARS-CoV-2 pseudoknot had predicted multiple different folding topologies. To confirm the existence of these predicted conformations, the researchers performed single-molecule optical tweezers measurements, using a hybrid DNA–RNA sample with a dual-beam optical tweezers setup (as shown in Figure 1A,B). In the context of constant velocity measurements, two unfolding patterns were identified. Approximately 80% of the traces showed a single unfolding event with a total contour length change of ∼35.6 nm, corresponding to the predicted unfolding size of the pseudoknot of 34.7–36.5 nm. Conversely, the remaining 20% of the traces only showed a contour length change of ∼25 nm, indicating that around 1/5th of the folded molecules failed to adopt the pseudoknot structure. This provides insight into the heterogeneous folding landscape of the SARS-CoV-2 pseudoknot, underscoring the complexity and inherent diversity in its structural dynamics.

**Figure 4.**
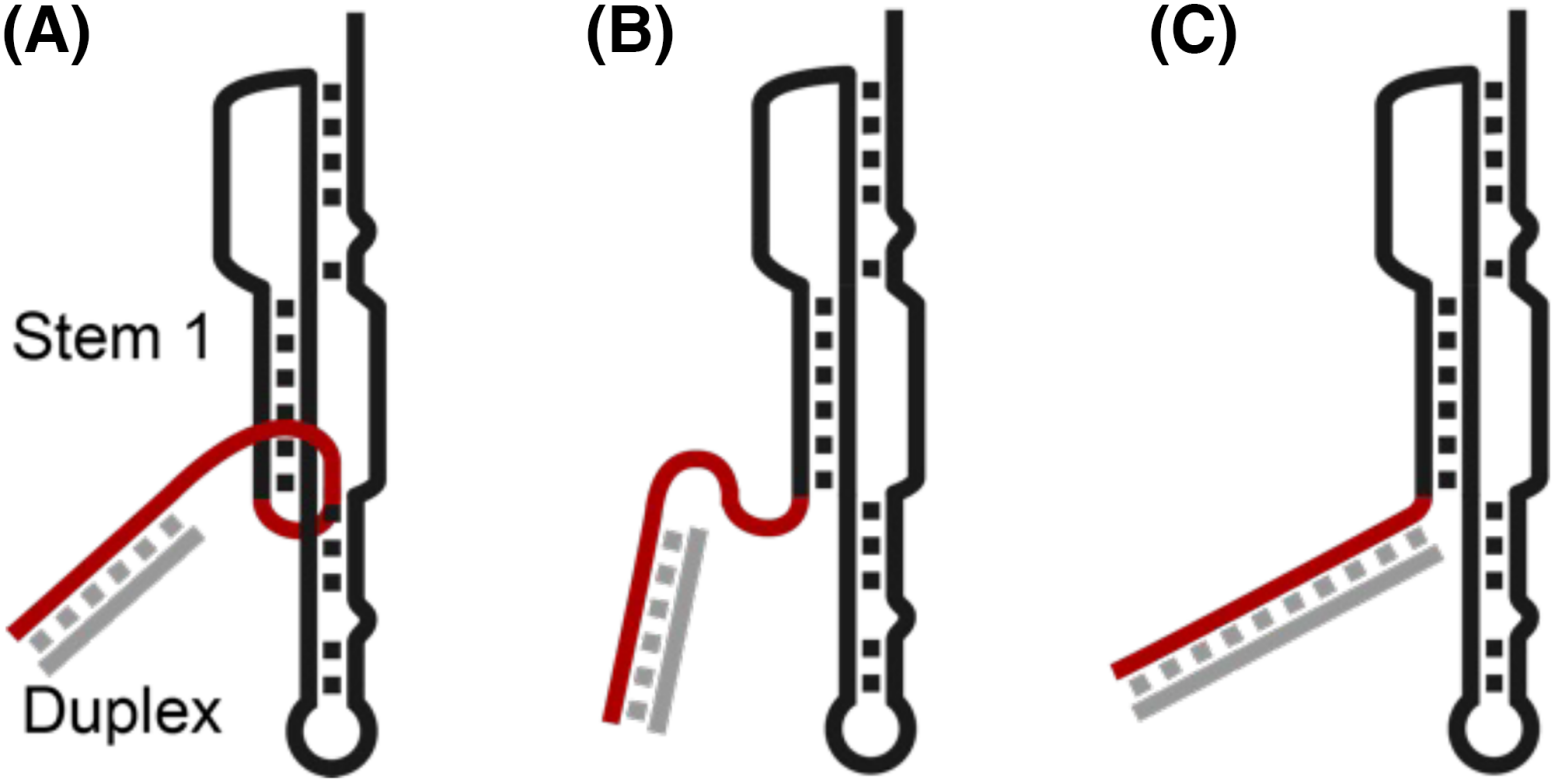
Schematic of the SARS-CoV-2 pseudoknot, RNA in black, 5′ side of RNA in red, DNA in grey. (**A**) Native structure where 5′ side is threaded inside of the pseudoknot, causing ribosomal frameshifting. Stem 1 and duplex labelled. (**B**) 5′ is unthreaded. (**C**) Extended RNA/DNA duplex blocking the ability to cause threaded pseudoknot.

To further look at the majority 80% of the traces, the forces required to cause the unfolding events were combined yielding a histogram with two discernible distributions. A high force distribution, unfolding at ∼30 pN, and a smaller distribution ∼16 pN were identified. It was hypothesised that the unfolding events of the higher forces belong to the fold in which the 5′ strand is threaded through the pseudoknot, while the lower force unfolding events correspond to an un-knotted structure (Figure 4A,B, respectively). This hypothesis was tested by extending the complementary DNA handle on the 5′-side towards the pseudo-knotted region to disrupt the knot formation. The closer proximity of the duplexed RNA/DNA was anticipated to reduce the chances of 5′ threading due to steric hindrance, reducing the overall mechanical stability of the structure. By decreasing the distance between the duplex region and stem 1 of the construct, the authors were able to show an increase in the low-force distribution, showing that the higher force distribution belonged to the pseudoknot with 5′ threading (Figure 4C).

It is noteworthy that the junction region of the pseudoknot had previously been identified as a potential target for binding small molecules making it a therapeutic target [33]. This study delineated two potential distinct RNA conformations that could be targeted, either by locking the pseudoknot into the 5′ threaded or the non-threaded conformation, both would greatly disturb the function of the coronavirus. This study reveals promising avenues for therapeutic interventions by strategically modulating the structural dynamics of the pseudoknot with the aim of mitigating viral pathogenicity.

**Figure 5.**
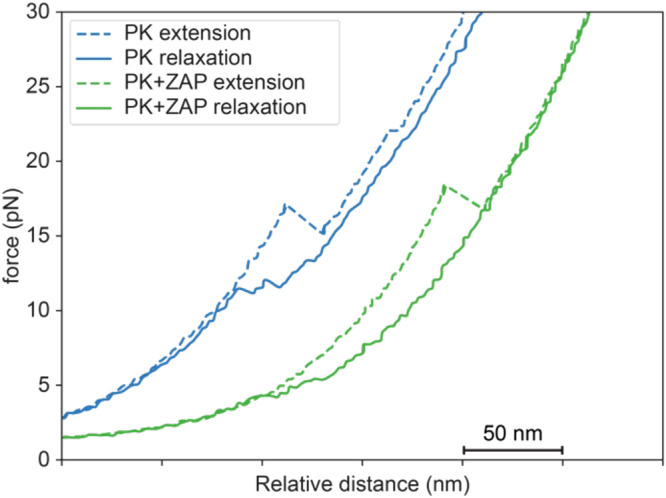
Extension and relaxation curves for full SARS-CoV-2 pseudoknot. Extension and relaxation curves for full SARS-CoV-2 pseudoknot [17]. A single unfolding event is visible under both conditions. However, during relaxation, the pseudoknot does not refold when ZAP is present.

In a similar study, Zimmer et al. [17] used optical tweezers to study the same frameshifting pseudoknot, with specific focus on the protein–RNA interactions that govern its functionality. To probe for protein interactors, they used *in vitro*RNA-antisense capture and mass spectrometry-based screening. The results showed that the zinc-finger antiviral protein (ZAP-S) was a strong interaction partner with the RNA pseudoknot. Further study showed that ZAP-S inhibits the viral frameshifting in both *in vivo* and *in vitro* by 20-fold. One aspect that they looked more closely into was the structural and dynamic perturbations the pseudoknot underwent in the presence of ZAP-S. To investigate the influence of ZAP-S on the pseudoknot, optical tweezers experiments were conducted with the RNA pseudoknot in the presence and absence of ZAP-S. A series of truncated transcripts were also tested to further identify consequential changes. Intriguingly, while almost no significant changes were observed at the unfolding phase of the pseudoknot, there was a notable change during the refolding phase (Figure 5). The majority of the tested traces exhibited the absence of refolding in the presence of ZAP-S, indicating that ZAP-S prevents the pseudoknot from folding into its native form. Additional analyses were performed with different segments of the pseudoknot RNA, demonstrating that ZAP-S serves as a potent inhibitor of the coronavirus frameshifting process.

### The Zika pseudoknot

**Figure 6.**
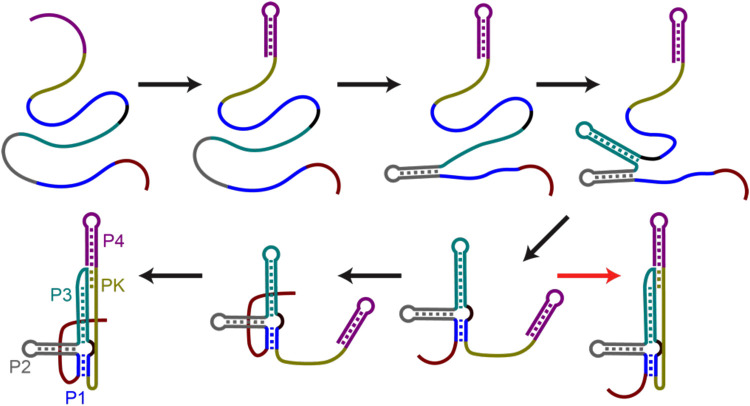
Folding pathway of the Zika pseudoknot. Black arrows indicate proper folding where the single-stranded 5′ side slips through before the pseudoknot is formed (pseudoknot PK in yellow). Red arrows indicate misfolding where 5′ side has not passed through.

Numerous human pathogens, including West Nile virus (WNV), dengue virus (DENV), Zika virus (ZIKV) and yellow fever virus (YFV), belong to the *Flaviviridae* family of viruses. These are small, enveloped viruses with a single-stranded RNA genome of ∼11 kb. This RNA genome comprises a 5′ untranslated region (5′-UTR), an open reading frame (ORF) and a 3′-UTR [34]. In the mid-2000s, an intriguing feature of flaviviruses was discovered — a structured region in the 3′-UTR of its RNA, conferring resistance to the XRN1 exonuclease [35]. This structure consists of four double-stranded RNA helices (labelled P1–P4 in Figure 6), and, analogous to SARS-COV-2, features a pseudoknot which provides the resistance against exonuclease degradation. In both cases, the resistance is hypothesised to originate from the high mechanical stability of the pseudoknot structure; however, it was not until 2021 when M. Zhao in the Woodside lab investigated this directly using single-molecule optical tweezers [36].

In their unfolding data, they found that the Zika pseudoknot exhibited unprecedently elevated unfolding forces compared with any previously observed RNA structures [36], close to the forces required for double-stranded DNA overstretching (∼65 pN). These high unfolding forces overlapping with the overstretching transition of the DNA handles made the accurate identification of unfolding events challenging. However, by increasing the waiting time in the high force state, the RNA molecule still completely unfolds. The complete unfolding of the molecule was validated by observing the expected refolding steps upon relaxation of the molecule. The majority of the force–extension curves showed that the molecule was in a strong, structured form, while 20% of the force–extension curves did not show this extreme mechanical resistance. Overall, four different pathways were identified: 80% of the time, the RNA molecule would fold into its native knotted pseudoknot, 4% of instances the pseudoknot would close before the 5′ end could thread through, 15% of the time, no threading or pseudoknot could form, resulting in an unconstrained 5′ end and the remaining 1% observed was when the 5′ end would form complementary binding to the pseudo-knotted region. All of this was deduced from the length changes observed in the force–extension curves and through measurements performed in the presence of antisense oligonucleotides to disrupt the pseudoknot formation.

Changing the waiting time at zero force between successive pulls did not result in a significant change in the percentage of threading of the 5′ end, indicating that the threading and pseudoknot formation happens rapidly and at low forces. Threading was, therefore, not observable in the force–extension curves, except indirectly, in the resulting high mechanical stability.

Finally, this study showed that both the pseudoknot and the contacts stabilising the threaded 5′ end were indispensable for the high force resistance of the knot. The removal of a single 5′-end contact resulted in a low-force knot, which did not exhibit exonuclease resistance, characteristic of the higher force scenario. Overall, this work unveils insights into the structural prerequisites that may govern the resistance against exonuclease degradation.

### The TAR hairpin of HIV

Reverse transcription, a pivotal step in the life cycle of the human immunodeficiency virus (HIV), is a process whereby the single-stranded (ss)RNA genome is converted into double-stranded (ds)DNA. Orchestrated by RNA-dependent DNA polymerases, also known as reverse transcriptases [37], this process is indispensable for viral genome integration and is an essential step in the HIV life cycle.

**Figure 7.**
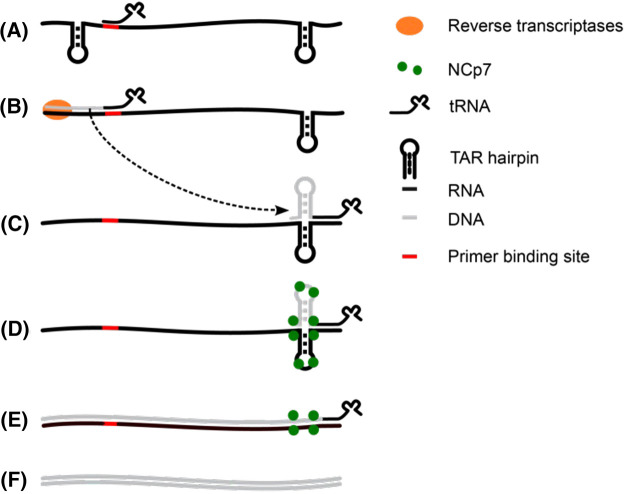
The six steps of minus-strand transfer in HIV. (**A**) tRNA binds to the primer binding site, on the 5′ side of the HIV genomic RNA. (**B**) Reverse transcriptase extends the tRNA. (**C**) Extended tRNA is denatured from the 5′ side and anneal to complementary 3′ region of genomic RNA. (**D**) NCp7 disrupts both TAR hairpins, which is needed for reverse transcriptase to finish full strand reverse transcription. (**E**) Reverse transcriptase completes DNA transcription. (**F**) Polymerase finishes dsDNA.

The HIV-1 RNA genome has an unusually shaped hairpin denoted the transactivation response (TAR) hairpin, located at both the 5′ and 3′ ends of the genome, which plays a key role in the reverse transcription process. The TAR hairpin structure is relatively simple, compared with the previously described RNA structures, it comprises 59 bases arranged to form a hairpin with three bulges of which two are a single nucleotide and two mismatches are within the stem. Despite its simple structure, it has been linked to multiple functions in the virus life cycle, underscoring its indispensability. One essential function of the TAR hairpin is aiding in the process of minus-strand transfer (Figure 7). During minus-strand transfer, the genomic RNA is transcribed into dsDNA and then integrated into the infected host genome.

The minus-strand transfer process starts by the annealing of a host tRNA to the primer binding site, close to the 5′ start of the viral genome. This event triggers the recruitment of the reverse transcriptase, which elongates the tRNA with ssDNA. The concomitant RNase H activity of the reverse transcriptase orchestrates the RNA, causing the elongated tRNA to dissociate and rebind to a complementary region on the 3′ side of the viral genome. However, here full reverse transcription is blocked by the TAR hairpin. Facilitating the unravelling of this halt in transcriptional activity is the nucleocapsid protein NCp7, a chaperone that destabilises the TAR hairpin, allowing for the full transcription and creation of viral dsDNA that can be incorporated into the host. NCp7 is a subdomain of the group-specific antigen (Gag) protein, and needs to be cleaved off from Gag to mature and function optimally [29]. However, it is unclear why NCp7 alone is more effective than when it is part of Gag, i.e. why Gag cleavage is an essential prerequisite for reverse transcription. This question was addressed by McCauley et al. [38], using a combination of optical tweezer experiments and computational modelling. Experiments were performed using a single-laser optical tweezers setup, where one bead is held in place using a micropipette, and the other is trapped by a moveable laser. Computer models, notably the widely used mfold nucleic acid folding and hybridisation prediction webserver, were used to computationally chart detailed energy landscapes of the TAR hairpin region.

Initial constant velocity unfolding and refolding experiments without Gag or NCp7 showed that ∼11 pN of force was imperative for the unravelling of a single TAR hairpin, resulting in a 10.9 nm length change. As mentioned before, the TAR hairpin consists of 59 bases, however, only a 48 base extension was observed, attributed to the low-force destabilisation of the lower part of the TAR stem, which could not be detected by the optical tweezers setup used.

In the presence of either NCp7 or Gag, the observed number of bases that unfold does not change significantly, meaning that the binding of either molecule to the RNA exerts negligible structural influence on the hairpin. However, the authors did find that the energy required to unfold the hairpin does change. In the absence of either NCp7 or Gag, the RNA hairpin requires 42 *k_B_T* (103.9 kJ/mol), whereas the presence of NCp7 reduced the energy barrier to 28.3 *k_B_T* (70.0 kJ/mol) and with Gag to 28.9 *k_B_T* (71.5 kJ/mol). The observed energy for unfolding in the presence of either of the two chaperones remains comparable, which means that energy considerations alone cannot explain why NCp7 is required for efficient minus-strand transfer.

When looking at the opening rate of the hairpin, which is defined as the frequency the hairpin opens per second, a notable difference is observed. The hairpin alone without any chaperones present is calculated to be 0.7 × 10^−8 ^s^−1^. In the presence of Gag, this remains relatively consistent, at 10 × 10^−8 ^s^−1^, however, in the presence of NCp7, the TAR hairpin has a significantly faster opening rate of 1.2 × 10^−4 ^s^−1^. McCauley et al. ascribe this difference to the interplay between Gag and NCp7. While the full-length Gag still contains NCp7 which can bind to, and destabilise, the TAR hairpin, it is significantly larger in size meaning that it can bind at fewer sites and at slower rates, having a diminished impact on the restructuring of the TAR hairpin.

## Conclusion and outlook

Non-coding RNAs, owing to their crucial roles in numerous diseases, represent a key frontier for therapeutic development [39]. This is emphasised and exemplified by the impact of the transformative efforts that led to mRNA vaccine development during the SARS-CoV-2 pandemic. RNA is involved in a legion of different steps in the life cycles of viruses, therefore, positioning ncRNA and the mechanisms they govern as promising avenues for novel treatments. However, the highly dynamic nature of RNA structure poses a challenge to fully unravelling these mechanisms using conventional methodologies [40].

The use of optical tweezers has enabled a detailed characterisation of RNA structures and interactions, through the measurement of their mechanical, thermodynamic and kinetic properties. Nevertheless, a huge number of disease-relevant RNA structures remain largely unexplored using these cutting-edge techniques. Furthermore, single-molecule *in vitro* measurements may of course be limited in their applicability, since RNA exists in the crowded environment of the cell and in the presence of multiple interacting partners — an intricate scenario challenging to replicate in a single-molecule optical tweezers setup. In light of these challenges, by building up the complexity of the measurement conditions, for example, by incorporating crowding agents, appropriate buffer conditions, and by systematically integrating interacting proteins, significant progress can be made towards bridging the gap. This approach unfolds as a promising strategy to gain valuable insights into the multiple roles of RNA structural dynamics in the context of disease.

## Perspectives

- RNA structure and conformational dynamics are recognised as pivotal determinants in various pathological conditions. Despite the limited number of experimental methodologies capable of elucidating real-time alterations in RNA structure, especially in response to diverse stimuli, recent advances in single-molecule investigations employing optical tweezers present a novel avenue for accessing and deciphering this invaluable information.
- The primary method that is used uniquely to study RNA structure, selective SHAPE has been revolutionary in providing access to a wealth of new data about RNA structure in cells. However, SHAPE does not provide direct access to the studied RNA molecules, rather it provides an indirect readout of their states, consequently precluding any real-time probing of their structural dynamics.
- The use of optical tweezers to study RNA has enabled a detailed characterisation of RNA structures and interactions, through measurement of their structural, thermodynamic and kinetic properties. Despite these advancements, a huge number of disease-relevant RNA structures are yet to be explored using these techniques. By providing an overview of what is currently possible and providing suggestions for bridging the gap between *in vitro* and *in vivo* RNA structure research, we aim to push this important research area of understanding the landscape of RNA structure, dynamics and biology forward.

## Open Access

Open access for this article was enabled by the participation of the University of Groningen in an all-inclusive *Read & Publish* agreement with Portland Press and the Biochemical Society under a transformative agreement with Individual.

## Abbreviations

cryo-EM: cryo-electron microscopy
HIV: human immunodeficiency virus
ncRNA: non-coding RNA
SHAPE: selective 2′-hydroxyl acylation analysed by primer extension
TAR: transactivation response
ZAP-S: zinc-finger antiviral protein
