# Correction: The application of single-molecule optical tweezers to study disease-related structural dynamics in RNA

**DOI:** 10.1042/BST20231232_COR

**Published:** 2026-02-05

**Authors:** Tycho Marinus, Toshana Foster, Katarzyna Tych

**Keywords:** optical tweezers, RNA, single-molecule

The authors of the original article “The application of single-molecule optical tweezers to study disease-related structural dynamics in RNA” 10.1042/BST20231232 would like to correct an error within one sentence of the “Basics of optical tweezers experiments” section (Page 3 of the online PDF). The word ‘biotin’ was incorrectly used instead of ‘streptavidin’ - the authors apologise for not spotting the mistake sooner.

The requested correction has been assessed and agreed by the Editorial Board. The authors declare that these corrections do not change the results or conclusions of their paper.

The corrected version of the sentence is presented here.

“These DNA handles are functionalised with biotin or digoxigenin moieties, which allows them to selectively bind to micron-sized streptavidin- or digoxigenin-coated beads, respectively [24].”

